# Poly[bis­{μ-*N*′-[(pyridin-4-yl)methyl­idene]benzohydrazidato}copper(II)]

**DOI:** 10.1107/S1600536813015882

**Published:** 2013-06-15

**Authors:** Qiong Wu, Da-Chi Chen, Chu-Yi Wu, Chang-Xiu Yan, Jian-Zhen Liao

**Affiliations:** a Department of Chemistry, Fuzhou University, Fuzhou, Fujian 350108, People’s Republic of China

## Abstract

In the title complex, [Cu(C_13_H_10_N_3_O)_2_]_*n*_, the copper(II) cation is located on a crystallographic inversion centre and adopts an elongated octa­hedral coordination geometry with the equatorial plane provided by *trans*-arranged bis-*N*,*O*-chelating acyl­hydrazine groups from two ligands and the apices by the N atoms of two pyridine rings belonging to symmetry-related ligands. The ligand adopts a *Z* conformation about the C=N double bond. The dihedral angle between the pyridine and phenyl rings is 2.99 (13)°. An intra­ligand C—H⋯N hydrogen bond is observed. In the crystal, each ligand bridges two adjacent metal ions, forming a (4,4) grid layered structure. π–π stacking inter­actions [centroid–centroid distances in the range 3.569 (4)–3.584 (9) Å] involving rings of adjacent layers result in the formation of a three-dimensional supra­molecular network.

## Related literature

For background to properties and applications of Schiff base–metal complexes, see: Schurig *et al.* (1980 ▶); Siddall *et al.* (1983 ▶); Maurya *et al.* (2005 ▶); Cozzi (2004 ▶); Liu *et al.* (2010 ▶). For the structures of related compounds, see: Yin (2008 ▶); Uçar *et al.* (2004 ▶); Sommerer *et al.* (1998 ▶); Moya-Hernández *et al.* (2003 ▶). 
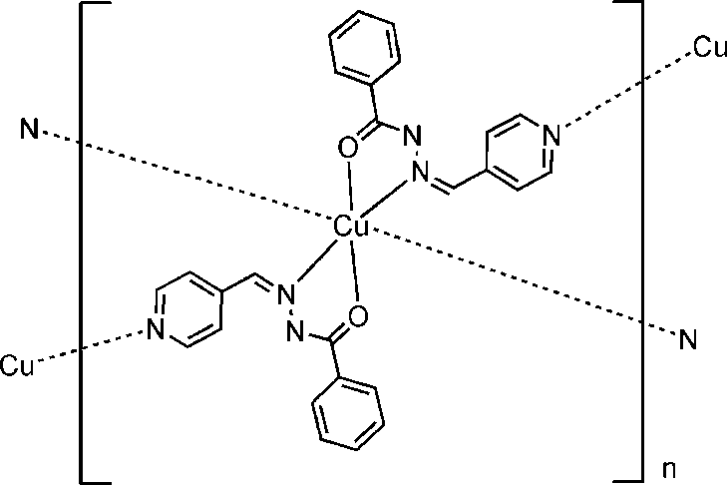


## Experimental

### 

#### Crystal data

[Cu(C_13_H_10_N_3_O)_2_]
*M* *_r_* = 512.02
Monoclinic, 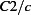

*a* = 12.288 (3) Å
*b* = 13.349 (3) Å
*c* = 14.244 (3) Å
β = 113.39 (3)°
*V* = 2144.5 (10) Å^3^
*Z* = 4
Mo *K*α radiation
μ = 1.06 mm^−1^
*T* = 173 K
0.40 × 0.20 × 0.12 mm

#### Data collection

Rigaku Mercury CCD area-detector diffractometer
Absorption correction: multi-scan (*CrystalClear*; Rigaku, 2007 ▶) *T*_min_ = 0.843, *T*_max_ = 1.000
10356 measured reflections
2460 independent reflections
2033 reflections with *I* > 2σ(*I*)
*R*_int_ = 0.035

#### Refinement

*R*[*F* ^2^ > 2σ(*F* ^2^)] = 0.033
*wR*(*F* ^2^) = 0.080
*S* = 1.05
2460 reflections
160 parameters
H-atom parameters constrained
Δρ_max_ = 0.33 e Å^−3^
Δρ_min_ = −0.19 e Å^−3^

### 

Data collection: *CrystalClear*; cell refinement: *CrystalClear*; data reduction: *CrystalClear*; program(s) used to solve structure: *SHELXS97* (Sheldrick, 2008 ▶); program(s) used to refine structure: *SHELXL97* (Sheldrick, 2008 ▶); molecular graphics: *SHELXTL* (Sheldrick, 2008 ▶); software used to prepare material for publication: *SHELXL97*.

## Supplementary Material

Crystal structure: contains datablock(s) I, global. DOI: 10.1107/S1600536813015882/rz5068sup1.cif

Structure factors: contains datablock(s) I. DOI: 10.1107/S1600536813015882/rz5068Isup2.hkl

Additional supplementary materials:  crystallographic information; 3D view; checkCIF report

## Figures and Tables

**Table 1:** Hydrogen-bond geometry (Å, °)

*D*—H⋯*A*	*D*—H	H⋯*A*	*D*⋯*A*	*D*—H⋯*A*
C10—H10⋯N1	0.95	2.32	2.907 (3)	120

**Fig. 1.**
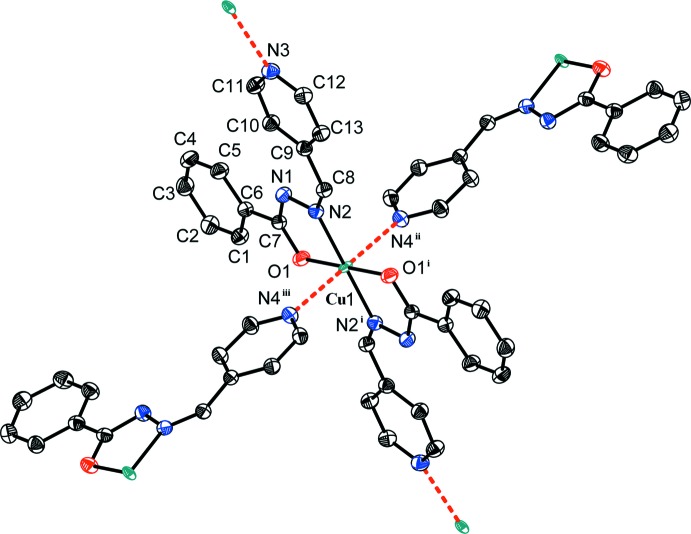
The molecular structure of the title compound with displacement ellipsoids drawn at the 30% probability level. H atoms are omitted for clarity. Symmetry codes: (i) 0.5 - x, 0.5 - y, -z; (ii) 0.5 - x, 0.5 + y, 0.5 - z; (iii) x, -y, -0.5 + z.

**Fig. 2.**
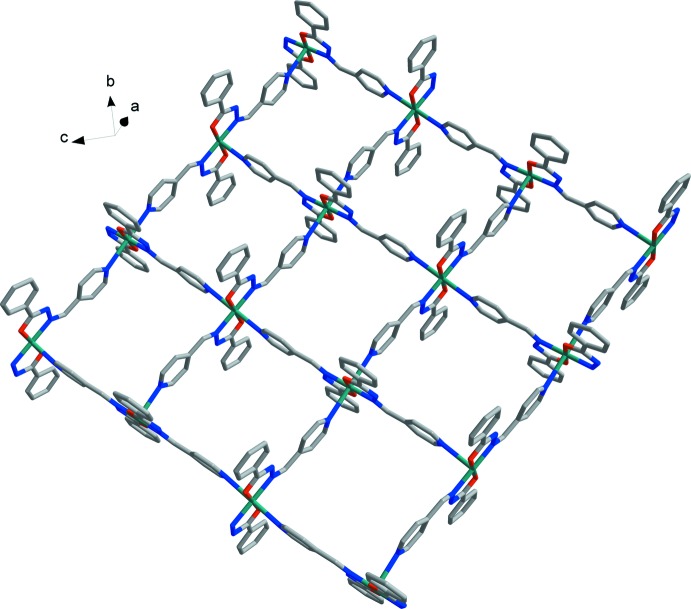
Capped sticks diagram of the two-dimensional grid in the title compound.

**Fig. 3.**
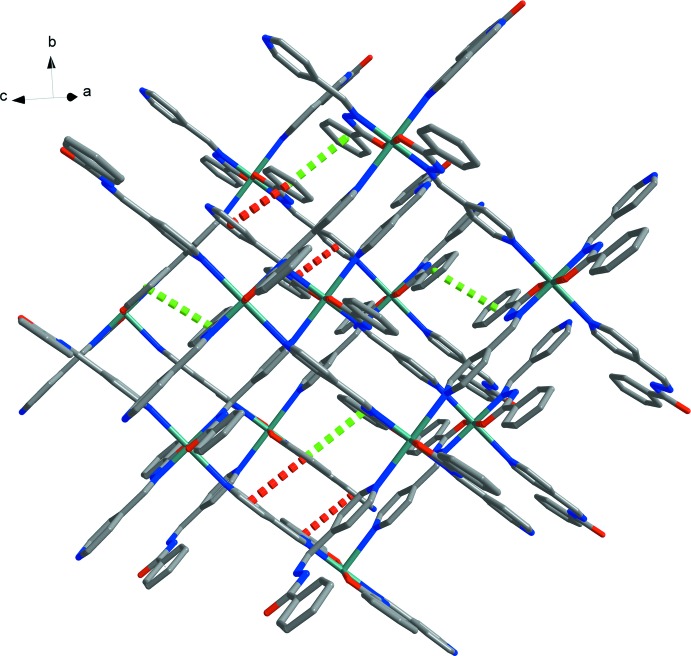
Packing diagram of the title compound, showing the π-π stacking interactions between phenyl rings (green dashed lines) or between phenyl and pyridine rings (red dashed lines). H atoms are omitted for clarity.

## supplementary crystallographic information

### Comment

Schiff bases and their metal compounds were widely synthesized in recent years and characterized for their wide range of applications as biocides and homogeneous catalysts in industry (Schurig *et al.*, 1980; Siddall *et al.*, 1983; Maurya *et al.*, 2005). In previous reports, most of the Schiff base coordination compounds were oligomers such as zero-dimensional complexes with catalytic (Cozzi, 2004) or magnetic properties (Liu, *et al.*, 2010). In this paper, the structure of a new two-dimensional polymeric copper(II) coordination compound is reported.

The asymmetric unit of the title complex contains one copper(II) ion located on an inversion centre and one deprotonated *N*'-(pyridin-4-ylmethyl) benzohydrazide ligand. The metal ion adopts a significantly elongated octahedral coordination geometry provided by four ligands (Fig. 1). The equatorial plane is occupied by two *trans*-arranged bis-N,*O*-chelating acylhydrazine groups from two ligands, while the axial positions are occupied by two N atoms of pyridine rings from other two ligands. In the equatorial plane, the Cu—O and Cu—N bond lengths are 1.942 (6) Å and 2.004 (4) Å, respectively, which are similar to those reported in the literature for related compounds (Yin 2008; Uçar *et al.*, 2004; Sommerer *et al.*, 1998). The apical Cu—N distances (2.578 (8) Å) are slightly longer than a common stretched Cu—N distance (Moya-Hernández *et al.*, 2003), generating an elongated octahedral coordination geometry typically attributed to the Jahn-Teller effect. The ligand is approximately planar (maximum deviation from the least square plane is 0.0473 (13) Å for atom O1) and chelates to the copper atom to form a five-numbered ring (Cu1/O1/C7/N1/N2). The dihedral angle formed by the pyridine and phenyl rings is 2.99 (13)°. An intraligand C—H···N hydrogen bond is present (Table 1). In the crystal, each ligand bridges two adjacent metal ions (Fig. 2), meanwhile each copper atom is coordinated with four ligands to form a (4, 4) grid layered structure. Adjacent layers are further connected *via*π···π stacking interactions between benzene rings [*Cg*1···*Cg*1^i^ =3.584 (9) Å; *Cg*1 is the centroid of the C1–C6 ring; symmetry code: (i) -1/2 - *x*, 1/2 - *y*, -*z*] and benzene and pyridine ring [*Cg*1···*Cg*2^ii^ = 3.569 (4) Å; *Cg*2 is the centroid of the N1/C9–C13 ring; symmetry code: (ii) -*x*, -*y*, -*z*], forming a three-dimensional supramolecular structure (Fig. 3).

### Experimental

A mixture of *N*'-(pyridin-4-ylmethyl)benzohydrazide (0.045 g, 0.02 mmol), Cu(CH_3_COO)_2_.4H_2_O (0.026 g, 0.01 mmol) in ethanol (6 mL) was stirred for 40 minutes and then heated in a 25 mL Teflon-lined autoclave at 100°C for 3 days, followed by cooling to room temperature. The resulting mixture was washed with water, and brown crystals were collected and dried in air. Yield: 40% (based on Cu).

### Refinement

All H atoms were placed in idealized positions using a riding-model approximation, with C—H = 0.95 Å and *U*_iso_(H) = 1.2*U*_eq_(C).

### Crystal data

**Table:**

[Cu(C_13_H_10_N_3_O)_2_]	*Z* = 4
*M**_r_* = 512.02	*F*(000) = 1052
Monoclinic, *C*2/*c*	*D*_x_ = 1.586 Mg m^−^^3^
Hall symbol: -C 2yc	Mo *K*α radiation, λ = 0.71073 Å
*a* = 12.288 (3) Å	µ = 1.06 mm^−^^1^
*b* = 13.349 (3) Å	*T* = 173 K
*c* = 14.244 (3) Å	Block, brown
β = 113.39 (3)°	0.40 × 0.20 × 0.12 mm
*V* = 2144.5 (10) Å^3^	

### Data collection

**Table:**

Rigaku Mercury CCD area-detector diffractometer	2460 independent reflections
Radiation source: fine-focus sealed tube	2033 reflections with *I* > 2σ(*I*)
Graphite monochromator	*R*_int_ = 0.035
ω scan	θ_max_ = 27.5°, θ_min_ = 3.1°
Absorption correction: multi-scan (*CrystalClear*; Rigaku, 2007)	*h* = −15→15
*T*_min_ = 0.843, *T*_max_ = 1.000	*k* = −17→17
10356 measured reflections	*l* = −18→18

### Refinement

**Table:**

Refinement on *F*^2^	Primary atom site location: structure-invariant direct methods
Least-squares matrix: full	Secondary atom site location: difference Fourier map
*R*[*F*^2^ > 2σ(*F*^2^)] = 0.033	Hydrogen site location: inferred from neighbouring sites
*wR*(*F*^2^) = 0.080	H-atom parameters constrained
*S* = 1.05	*w* = 1/[σ^2^(*F*_o_^2^) + (0.038*P*)^2^ + 1.4497*P*] where *P* = (*F*_o_^2^ + 2*F*_c_^2^)/3
2460 reflections	(Δ/σ)_max_ < 0.001
160 parameters	Δρ_max_ = 0.33 e Å^−^^3^
0 restraints	Δρ_min_ = −0.19 e Å^−^^3^

### Special details

**Table:**

Geometry. All e.s.d.'s (except the e.s.d. in the dihedral angle between two l.s. planes) are estimated using the full covariance matrix. The cell e.s.d.'s are taken into account individually in the estimation of e.s.d.'s in distances, angles and torsion angles; correlations between e.s.d.'s in cell parameters are only used when they are defined by crystal symmetry. An approximate (isotropic) treatment of cell e.s.d.'s is used for estimating e.s.d.'s involving l.s. planes.
Refinement. Refinement of *F*^2^ against ALL reflections. The weighted *R*-factor *wR* and goodness of fit *S* are based on *F*^2^, conventional *R*-factors *R* are based on *F*, with *F* set to zero for negative *F*^2^. The threshold expression of *F*^2^ > σ(*F*^2^) is used only for calculating *R*-factors(gt) *etc*. and is not relevant to the choice of reflections for refinement. *R*-factors based on *F*^2^ are statistically about twice as large as those based on *F*, and *R*- factors based on ALL data will be even larger.

### Fractional atomic coordinates and isotropic or equivalent isotropic displacement parameters (Å^2^)

**Table:**

	*x*	*y*	*z*	*U*_iso_*/*U*_eq_	
Cu1	0.2500	0.2500	0.0000	0.03006 (12)	
O1	0.07807 (11)	0.24346 (9)	−0.06638 (10)	0.0309 (3)	
N3	0.27033 (14)	−0.10391 (12)	0.38950 (12)	0.0319 (4)	
N2	0.21900 (13)	0.15245 (10)	0.09372 (11)	0.0246 (3)	
N1	0.09996 (13)	0.13652 (11)	0.06875 (12)	0.0278 (3)	
C13	0.38186 (16)	−0.00109 (14)	0.32256 (15)	0.0323 (4)	
H13	0.4576	0.0211	0.3281	0.039*	
C10	0.17175 (16)	0.00125 (14)	0.24281 (15)	0.0321 (4)	
H10	0.0988	0.0242	0.1923	0.038*	
C11	0.17304 (17)	−0.06719 (15)	0.31632 (15)	0.0346 (4)	
H11	0.0988	−0.0896	0.3143	0.042*	
C9	0.27962 (16)	0.03584 (13)	0.24435 (14)	0.0265 (4)	
C7	0.03622 (16)	0.18736 (13)	−0.01562 (14)	0.0258 (4)	
C12	0.37330 (17)	−0.06991 (15)	0.39198 (15)	0.0329 (4)	
H12	0.4444	−0.0941	0.4440	0.039*	
C1	−0.16555 (18)	0.23105 (14)	−0.13975 (16)	0.0339 (4)	
H1	−0.1296	0.2705	−0.1751	0.041*	
C6	−0.09470 (16)	0.17841 (13)	−0.05313 (14)	0.0278 (4)	
C8	0.29530 (15)	0.10671 (13)	0.17138 (14)	0.0265 (4)	
H8	0.3756	0.1211	0.1839	0.032*	
C3	−0.34020 (18)	0.16951 (16)	−0.12463 (17)	0.0405 (5)	
H3	−0.4241	0.1675	−0.1482	0.049*	
C4	−0.27127 (19)	0.11491 (17)	−0.03975 (18)	0.0418 (5)	
H4	−0.3079	0.0743	−0.0058	0.050*	
C2	−0.28764 (18)	0.22705 (15)	−0.17542 (17)	0.0389 (5)	
H2	−0.3352	0.2638	−0.2347	0.047*	
C5	−0.14871 (17)	0.11890 (15)	−0.00365 (16)	0.0362 (4)	
H5	−0.1015	0.0810	0.0549	0.043*	

### Atomic displacement parameters (Å^2^)

**Table:**

	*U*^11^	*U*^22^	*U*^33^	*U*^12^	*U*^13^	*U*^23^
Cu1	0.02433 (17)	0.0392 (2)	0.02625 (18)	−0.00444 (14)	0.00965 (13)	0.01200 (14)
O1	0.0302 (6)	0.0357 (7)	0.0279 (7)	−0.0040 (6)	0.0128 (6)	0.0066 (5)
N3	0.0378 (9)	0.0309 (8)	0.0280 (9)	0.0025 (7)	0.0141 (7)	0.0053 (6)
N2	0.0270 (7)	0.0247 (7)	0.0247 (8)	−0.0015 (6)	0.0130 (6)	−0.0002 (6)
N1	0.0257 (7)	0.0301 (8)	0.0282 (8)	−0.0026 (6)	0.0113 (6)	0.0050 (6)
C13	0.0284 (9)	0.0358 (10)	0.0337 (11)	0.0008 (8)	0.0136 (8)	0.0044 (8)
C10	0.0285 (9)	0.0346 (9)	0.0307 (10)	0.0012 (8)	0.0090 (8)	0.0077 (8)
C11	0.0315 (10)	0.0369 (10)	0.0363 (11)	−0.0004 (8)	0.0144 (9)	0.0088 (8)
C9	0.0309 (9)	0.0235 (8)	0.0262 (9)	0.0025 (7)	0.0125 (8)	0.0013 (7)
C7	0.0314 (9)	0.0238 (8)	0.0247 (9)	−0.0023 (7)	0.0139 (8)	−0.0017 (7)
C12	0.0320 (9)	0.0353 (10)	0.0289 (10)	0.0065 (8)	0.0094 (8)	0.0072 (8)
C1	0.0352 (10)	0.0333 (10)	0.0337 (11)	−0.0001 (8)	0.0143 (9)	0.0064 (8)
C6	0.0308 (9)	0.0272 (9)	0.0276 (10)	−0.0028 (7)	0.0139 (8)	−0.0006 (7)
C8	0.0279 (9)	0.0265 (8)	0.0272 (9)	0.0003 (7)	0.0133 (8)	0.0017 (7)
C3	0.0301 (10)	0.0448 (12)	0.0471 (13)	−0.0040 (9)	0.0159 (9)	−0.0033 (9)
C4	0.0391 (11)	0.0462 (12)	0.0455 (12)	−0.0106 (10)	0.0227 (10)	0.0031 (10)
C2	0.0340 (10)	0.0396 (11)	0.0382 (12)	0.0031 (9)	0.0092 (9)	0.0068 (8)
C5	0.0350 (10)	0.0402 (11)	0.0339 (11)	−0.0026 (9)	0.0141 (9)	0.0080 (8)

### Geometric parameters (Å, º)

**Table:**

Cu1—O1^i^	1.9440 (15)	C11—H11	0.9500
Cu1—O1	1.9440 (15)	C9—C8	1.474 (2)
Cu1—N2^i^	2.0066 (14)	C7—C6	1.485 (2)
Cu1—N2	2.0066 (14)	C12—H12	0.9500
O1—C7	1.282 (2)	C1—C2	1.381 (3)
N3—C11	1.329 (2)	C1—C6	1.386 (3)
N3—C12	1.331 (2)	C1—H1	0.9500
N2—C8	1.285 (2)	C6—C5	1.393 (3)
N2—N1	1.378 (2)	C8—H8	0.9500
N1—C7	1.331 (2)	C3—C4	1.377 (3)
C13—C12	1.384 (3)	C3—C2	1.379 (3)
C13—C9	1.397 (3)	C3—H3	0.9500
C13—H13	0.9500	C4—C5	1.386 (3)
C10—C11	1.385 (3)	C4—H4	0.9500
C10—C9	1.395 (3)	C2—H2	0.9500
C10—H10	0.9500	C5—H5	0.9500
			
O1^i^—Cu1—O1	180.0	N1—C7—C6	116.68 (15)
O1^i^—Cu1—N2^i^	80.66 (6)	N3—C12—C13	123.25 (18)
O1—Cu1—N2^i^	99.34 (6)	N3—C12—H12	118.4
O1^i^—Cu1—N2	99.34 (6)	C13—C12—H12	118.4
O1—Cu1—N2	80.66 (6)	C2—C1—C6	120.98 (18)
N2^i^—Cu1—N2	180.00 (8)	C2—C1—H1	119.5
C7—O1—Cu1	110.72 (12)	C6—C1—H1	119.5
C11—N3—C12	116.40 (16)	C1—C6—C5	118.85 (17)
C8—N2—N1	119.06 (15)	C1—C6—C7	119.16 (16)
C8—N2—Cu1	127.92 (12)	C5—C6—C7	121.98 (17)
N1—N2—Cu1	113.01 (11)	N2—C8—C9	131.08 (16)
C7—N1—N2	109.77 (14)	N2—C8—H8	114.5
C12—C13—C9	120.31 (18)	C9—C8—H8	114.5
C12—C13—H13	119.8	C4—C3—C2	120.16 (19)
C9—C13—H13	119.8	C4—C3—H3	119.9
C11—C10—C9	118.73 (18)	C2—C3—H3	119.9
C11—C10—H10	120.6	C3—C4—C5	120.28 (19)
C9—C10—H10	120.6	C3—C4—H4	119.9
N3—C11—C10	124.95 (18)	C5—C4—H4	119.9
N3—C11—H11	117.5	C3—C2—C1	119.68 (19)
C10—C11—H11	117.5	C3—C2—H2	120.2
C10—C9—C13	116.34 (17)	C1—C2—H2	120.2
C10—C9—C8	126.20 (17)	C4—C5—C6	120.01 (19)
C13—C9—C8	117.45 (16)	C4—C5—H5	120.0
O1—C7—N1	125.68 (16)	C6—C5—H5	120.0
O1—C7—C6	117.64 (16)		
			
N2^i^—Cu1—O1—C7	−176.85 (12)	C11—N3—C12—C13	−0.8 (3)
N2—Cu1—O1—C7	3.15 (12)	C9—C13—C12—N3	−0.5 (3)
O1^i^—Cu1—N2—C8	−2.68 (16)	C2—C1—C6—C5	1.6 (3)
O1—Cu1—N2—C8	177.32 (16)	C2—C1—C6—C7	−178.12 (18)
O1^i^—Cu1—N2—N1	176.46 (11)	O1—C7—C6—C1	−0.6 (3)
O1—Cu1—N2—N1	−3.54 (11)	N1—C7—C6—C1	178.95 (17)
C8—N2—N1—C7	−177.64 (15)	O1—C7—C6—C5	179.64 (17)
Cu1—N2—N1—C7	3.13 (17)	N1—C7—C6—C5	−0.8 (3)
C12—N3—C11—C10	1.3 (3)	N1—N2—C8—C9	−0.2 (3)
C9—C10—C11—N3	−0.3 (3)	Cu1—N2—C8—C9	178.87 (14)
C11—C10—C9—C13	−1.0 (3)	C10—C9—C8—N2	0.8 (3)
C11—C10—C9—C8	178.68 (18)	C13—C9—C8—N2	−179.51 (18)
C12—C13—C9—C10	1.4 (3)	C2—C3—C4—C5	1.2 (3)
C12—C13—C9—C8	−178.30 (17)	C4—C3—C2—C1	−1.1 (3)
Cu1—O1—C7—N1	−2.6 (2)	C6—C1—C2—C3	−0.3 (3)
Cu1—O1—C7—C6	176.93 (12)	C3—C4—C5—C6	0.1 (3)
N2—N1—C7—O1	−0.4 (2)	C1—C6—C5—C4	−1.5 (3)
N2—N1—C7—C6	−179.92 (14)	C7—C6—C5—C4	178.24 (19)

Symmetry code: (i) −*x*+1/2, −*y*+1/2, −*z*.

### Hydrogen-bond geometry (Å, º)

**Table:**

*D*—H···*A*	*D*—H	H···*A*	*D*···*A*	*D*—H···*A*
C10—H10···N1	0.95	2.32	2.907 (3)	120
